# Preventing COVID-19 Outbreaks in Long-Term Care Facilities Through Preemptive Testing of Residents and Staff Members — Fulton County, Georgia, March–May 2020

**DOI:** 10.15585/mmwr.mm6937a4

**Published:** 2020-09-18

**Authors:** Carson T. Telford, Udodirim Onwubiko, David P. Holland, Kim Turner, Juliana Prieto, Sasha Smith, Jane Yoon, Wecheeta Brown, Allison Chamberlain, Neel Gandhi, Steve Williams, Fazle Khan, Sarita Shah

**Affiliations:** ^1^Office of Epidemiology, Fulton County Board of Health, Atlanta, Georgia; ^2^Rollins School of Public Health, Emory University, Atlanta, Georgia; ^3^Department of Medicine, Emory University, Atlanta, Georgia; ^4^Fulton County Government, Atlanta, Georgia.

 Long-term care facility (LTCF) residents are at particularly high risk for morbidity and mortality associated with infection with SARS-CoV-2, the virus that causes coronavirus disease 2019 (COVID-19), given their age and high prevalence of chronic medical conditions, combined with functional impairment that often requires frequent, close contact with health care providers, who might inadvertently spread the virus to residents (*1*,*2*). During March–May 2020 in Fulton County, Georgia, >50% of COVID-19–associated deaths occurred among LTCF residents, although these persons represented <1% of the population (*3*,*4*). Mass testing for SARS-CoV-2 has been an effective strategy for identifying asymptomatic and presymptomatic infections in LTCFs (*5*). This analysis sought to evaluate the timing at which mass testing took place in relation to the known presence of a COVID-19 infection and the resulting number of infections that occurred. In 15 LTCFs that performed facility-wide testing in response to an identified case, high prevalences of additional cases in residents and staff members were found at initial testing (28.0% and 7.4%, respectively), suggesting spread of infection had already occurred by the time the first case was identified. Prevalence was also high during follow-up, with a total of 42.4% of residents and 11.8% of staff members infected overall in the response facilities. In comparison, 13 LTCFs conducted testing as a preventive strategy before a case was identified. Although the majority of these LTCFs identified at least one COVID-19 case, the prevalence was significantly lower at initial testing in both residents and staff members (0.5% and 1.0%, respectively) and overall after follow-up (1.5% and 1.7%, respectively). These findings indicate that early awareness of infections might help facilities prevent potential outbreaks by prioritizing and adhering more strictly to infection prevention and control (IPC) recommendations, resulting in fewer infections than would occur when relying on symptom-based screening (*6*,*7*).

Facility-wide testing in LTCFs (i.e., skilled nursing, memory care, and assisted living facilities) in Fulton County began when the first COVID-19 LTCF outbreak was identified in March 2020. Because SARS-CoV-2 test kits and staffing capacity were limited, facility-wide testing at the beginning of the COVID-19 pandemic focused on LTCFs that already had at least one known confirmed case of COVID-19 in a resident or staff member; these initial cases were detected through testing of symptomatic persons. Testing was carried out 1–5 days after identification of the index case,* depending on Fulton County Board of Health (FCBOH) field testing team availability. Mass testing in LTCFs without known infections began on April 29^†^ when additional testing support was provided by the National Guard. A 1-day testing event for consenting residents and staff members was held at each LTCF during March 31–May 18 to identify the baseline infection prevalence, and symptom-based screening was conducted for 4 weeks thereafter to identify subsequent cases. Testing at 15 facilities was conducted in response to a confirmed SARS-CoV-2 infection identified through symptom-based screening; those tested at these 15 facilities were referred to as “the response group.” There were 13 LTCFs that conducted preemptive testing before any case had been identified^§^; those tested at these 13 facilities were referred to as “the preventive group.”

Trained health care staff members from FCBOH and the National Guard collected nasopharyngeal swab samples at 22 LTCFs. Samples were tested for SARS-CoV-2 by real-time reverse transcription–polymerase chain reaction (RT-PCR) at various Georgia laboratories; a confirmed COVID-19 case was defined as a positive RT-PCR test result. When test results were received from participating laboratories they were immediately reported to the facilities, and IPC guidance and implementation support was provided at sites where positive results were identified to mitigate further disease transmission (*6*). Six LTCFs (three in each group) contracted with private companies to collect nasopharyngeal swabs and perform RT-PCR testing; these results were reported to FCBOH. Sample collection, transportation, and testing were conducted in accordance with the most recent CDC guidelines (*8*). Staff members absent on the day of testing but who provided evidence of a positive RT-PCR test result were included in this analysis, and those employed and tested at multiple LTCFs were counted in each facility’s staff census and case count. One LTCF declined testing for all staff members. Each LTCF’s number and proportion of SARS-CoV-2–positive RT-PCR test results, hospitalizations, and deaths among residents and staff members were calculated based on the sum of cases identified through mass testing and throughout 4 weeks of follow-up symptom-based screening.^¶^ Fisher’s exact tests were used to test differences between facility groups; p-values <0.05 were considered to be statistically significant. This activity was reviewed by the Georgia Department of Public Health and determined to be consistent with public health surveillance as described in Title 45 Code of Federal Regulations 46.102(1)(2).

Overall, 5,671 persons from 28 LTCFs were tested, including 2,868 (50.6%) residents and 2,803 (49.4%) staff members. During the facility-wide testing events, 637 (11.2%) persons received positive test results for SARS-CoV-2, including 484 (16.9%) residents and 153 (5.5%) staff members.** At the end of the follow-up period, 348 additional positive SARS-CoV-2 test results were reported, for a total of 985 (17.4%) persons with positive SARS-CoV-2 test results (residents = 740 [25.8%]; staff members = 245 [8.7%]). At the time of initial testing, resident prevalence was 28.0% in the response group and 0.5% in the preventive group (p<0.01). Among staff members, prevalence at initial testing was 7.4% in the response group and 1.0% in the preventive group (p<0.01). Eight (61.5%) LTCFs in the preventive group reported at least one COVID-19 infection at the time of initial testing. After 4 weeks of follow-up, the overall resident prevalence was 42.4% and 1.5% in the response and preventive LTCFs, respectively, and prevalence among staff members was 11.8% and 1.7% in the response and preventive groups, respectively (p<0.001 for both residents and staff members) (Table).

**TABLE:** COVID-19 cases, hospitalizations, and deaths among long-term care facility residents and staff members — Fulton County, Georgia, March–May 2020

LTCF ID, date screened		Residents, no. (%)					Staff members, no. (%)				
		No. tested*	Cases identified through mass testing^†^	Total cases identified^§^	Hospitalized^¶^	Died^¶^	No. tested*	Cases identified through mass testing^†^	Total cases identified^§^	Hospitalized^¶^	Died^¶^
**Response group**^¶,^**											
1	3/31/20	176	36 (20.5)	106 (60.2)	18 (17.0)	21 (19.8)	74	22 (29.7)	40 (54.1)	0	0
2	4/3/20	63	32 (50.8)	50 (79.4)	17 (34.0)	15 (30.0)	81	15 (18.5)	32 (39.5)	6 (18.8)	0
3	4/5/20	69	14 (20.3)	17 (24.6)	4 (23.5)	2 (11.8)	135	9 (6.7)	11 (8.1)	0	0
4	4/8/20	67	45 (67.2)	49 (73.1)	16 (32.7)	10 (20.4)	56	27 (48.2)	31 (55.4)	2 (6.5)	0
5	4/11/20	38	12 (31.6)	16 (42.1)	4 (25.0)	2 (12.5)	61	7 (11.5)	13 (21.3)	0	0
6	4/13/20	78	6 (7.7)	10 (12.8)	2 (20.0)	6 (60.0)	199	2 (1.0)	12 (6.0)	0	0
7	4/15/20	112	40 (35.7)	45 (40.2)	5 (11.1)	1 (2.2)	116	13 (11.2)	17 (14.7)	0	0
8	4/16/20	88	17 (19.3)	20 (22.7)	3 (15.0)	1 (5.0)	126	6 (4.8)	7 (5.6)	2 (28.6)	0
9	4/19/20	167	104 (62.3)	117 (70.1)	20 (17.1)	10 (8.5)	130	10 (7.7)	16 (12.3)	0	0
10	4/22/20	96	24 (25.0)	24 (25.0)	5 (20.8)	3 (12.5)	104	3 (2.9)	4 (3.8)	0	0
11	4/28/20	196	39 (19.9)	50 (25.5)	4 (8.0)	3 (6.0)	150	4 (2.7)	4 (2.7)	0	0
12	4/30/20	196	10 (5.1)	48 (24.5)	6 (12.5)	2 (4.2)	252	2 (0.8)	3 (1.2)	0	0
13	5/7/20	94	8 (8.5)	28 (29.8)	8 (28.6)	4 (14.3)	75	2 (2.7)	4 (5.3)	0	0
14	5/11/20	81	39 (48.1)	46 (56.8)	6 (13.0)	6 (13.0)	106	6 (5.7)	10 (9.4)	1 (10.0)	0
15	5/14/20	184	52 (28.3)	97 (52.7)	26 (26.8)	23 (23.7)	279	16 (5.7)	26 (9.3)	3 (11.5)	0
**Total response**		**1,705**	**478 (28.0)**	**723 (42.4)**	**144 (19.9)**	**109 (15.1)**	**1944**	**144 (7.4)**	**230 (11.8)**	**14 (6.1)**	**0**
**Preventive group** ^††^											
16^§§^	4/2/20	287	1 (0.3)	1 (0.3)	0	0	270	0	0	0	0
17^¶¶^	4/29/20	102	1 (1.0)	1 (1.0)	0	0	0	0	0	0	0
18	5/5/20	26	1 (3.8)	4 (15.4)	4 (100.0)	3 (75.0)	8	0	0	0	0
19	5/6/20	64	0	0	0	0	64	0	0	0	0
20	5/11/20	73	0	0	0	0	46	1 (2.2)	2 (4.3)	0	0
21	5/13/20	78	1 (1.3)	1 (1.3)	1 (100.0)	0	100	0	1 (1.0)	0	0
22	5/18/20	46	0	0	0	0	43	0	0	0	0
23	5/27/20	35	0	0	0	0	19	0	0	0	0
24	5/27/20	48	0	6 (12.5)	0	0	76	6 (7.9)	10 (13.2)	0	0
25	5/28/20	218	1 (0.5)	2 (0.9)	0	0	100	2 (2.0)	2 (2.0)	1 (50.0)	1 (50.0)
26	5/29/20	87	1 (1.1)	2 (2.3)	0	0	97	0	0	0	0
27	5/29/20	1	0	0	0	0	30	0	0	0	0
28	5/29/20	98	0	0	0	0	6	0	0	0	0
**Total preventive**		**1,163**	**6 (0.5)**	**17 (1.5)**	**5 (29.4)**	**3 (17.6)**	**859**	**9 (1.0)**	**15 (1.7)**	**1 (6.7)**	**1 (6.7)**
**All facilities**		**2,868**	**484 (16.9)**	**740 (25.8)**	**149 (20.1)**	**112 (15.1)**	**2803**	**153 (5.5)**	**245 (8.7)**	**15 (6.1)**	**1 (0.4)**
p-value***		**—**	**<0.01**	**<0.01**	**0.36**	**0.73**	**—**	**<0.01**	**<0.01**	**1**	**0.06**

**Abbreviations:** COVID-19 = coronavirus disease 2019; LTCF = long-term care facility.

* Residents and staff members who consented and were present on the day of testing.

**^†^** Percentage among all persons tested.

^§^ Total cases identified through mass screening and 4 weeks of symptom-based screening.

^¶^ Percentage among persons with positive test results for SARS-CoV-2, the virus that causes COVID-19.

** LTCFs in which facility-wide COVID-19 testing was initiated in response to identification of the index case through symptom-based screening. Cases after the mass-testing event were identified using symptom-based screening.

^††^ LTCFs in which facility-wide COVID-19 testing was initiated before identification of a COVID-19 case. Cases after the mass-testing event were identified using symptom-based screening.

^§§^ The only preventive group LTCF that was tested by Fulton County Board of Health before supplemental testing support was added by the National Guard.

^¶¶^ Declined testing for staff members.

*** p-value results of Fisher’s exact test comparing all LTCFs in the preventive group to all LTCFs in the response group for the following indicators: COVID-19 diagnoses, hospitalizations and deaths.

Among the 985 persons who received a diagnosis of COVID-19 during and after the facility-wide testing, 164 (16.6%) required hospitalization, and 113 (11.5%) died. Most hospitalizations (158; 96.3%) occurred in the response group, but the proportion of residents hospitalized because of COVID-19 did not differ significantly between the response (19.9%) and preventive (29.4%) groups (p = 0.36). Similarly, the proportion of residents in the response group who died (15.1%) was similar to that in the preventive group (17.6%) (p = 0.73); however, 109 (96.5%) of all 113 deaths occurred in the response group LTCFs; only one death occurred in a staff member, and that was in the preventive group.

.

## Discussion

In this analysis of facility-wide SARS-CoV-2 testing and follow-up at 28 LTCFs in Fulton County, Georgia, SARS-CoV-2 infection was identified in 25.8% of residents and 8.7% of staff members. Facilities which conducted testing after a known, confirmed case of COVID-19 were found to have significantly higher proportions of infected residents and staff members at initial testing and at follow-up, suggesting spread had already occurred by the time the first case was identified. Importantly, even in LTCFs that tested residents and staff members preemptively before a known infection, at least one case was identified in the majority of these facilities. However, the initial prevalence was significantly lower and fewer cases occurred during follow-up, supporting the potential for early testing to prevent outbreaks when combined with IPC recommendations (*6*,*7*). Although this analysis assessed a single mass testing event and subsequent follow-up period to identify new cases, the Center for Medicare & Medicaid Services currently requires routine testing with a 48-hour turnaround of LTCF residents and staff members^††^ at varying frequencies contingent on the proportion of positive tests in the community of the facility (*9*).

The findings in this report are subject to at least four limitations. First, because LTCFs in the preventive group did not identify a COVID-19 case until later dates, they might have been at lower risk overall. Although risk for a COVID-19 outbreak at the beginning of the COVID-19 pandemic might have varied by facility, as of August 16, 2020, outbreaks continued to be reported in LTCFs where cases had not previously been identified, indicating that LTCFs not affected when COVID-19 was first introduced to Fulton County continued to be at risk for outbreaks. COVID-19 might have been introduced to some LTCFs in the response group before a shelter-in-place order was issued by the state of Georgia,^§§^ which prohibited LTCF resident visitation, although it was likely introduced to remaining LTCFs after the shelter-in-place order.^¶¶^ After the shelter-in-place order, the most likely mode of infection among residents was through exposure to an infected staff member, although several preventive group LTCFs reported COVID-19 cases in residents but not staff members. It is possible that these residents had been infected earlier in the pandemic but were asymptomatic and possibly no longer infectious at the time of testing (*10*). Second, guidance from CDC on IPC strategies was released on May 8, 2020, after some response group LTCFs were tested, possibly contributing to the lower prevalence of infection in LTCFs tested at later dates. Third, response group LTCFs were tested based on reports of COVID-19 cases and were not selected at random to provide a representative sample. Nonetheless, these facilities represented 48.3% of licensed LTCFs and 44.4% of the total bed capacity of LTCFs in Fulton County (*4*). Finally, identification of cases during the follow-up period relied on reporting from LTCFs to the FCBOH. Census lists provided by LTCFs and case reports from hospitals and medical examiners were used to identify and retroactively link unreported outcomes to their respective LTCF. Because follow-up used symptom-based screening, persons infected after mass testing who remained asymptomatic could not be identified, leading to potential underrepresentation of the total number of SARS-CoV-2 infections in both groups.

The COVID-19 pandemic has highlighted the vulnerability of residents and staff members of LTCFs. Findings from this analysis of facility-wide testing efforts in Fulton County suggest that active testing of LTCF residents and staff members can identify some COVID-19 cases early, guide IPC response, and reduce the spread of SARS-CoV-2 (*7*,*9*).

**Summary**

**What is already known about this topic?**

Residents of long-term care facilities (LTCFs) are at risk for severe COVID-19. Facility-wide testing, even in the absence of a reported COVID-19 case, can identify asymptomatic and presymptomatic infection in LTCFs.

**What is added by this report?**

LTCFs in which testing was conducted after a confirmed case of COVID-19 were found to have significantly higher proportions of infected residents and staff members at initial testing and after 4 weeks of follow-up compared with those testing as a preventive measure. The majority of LTCFs testing as a preventive measure identified an infection, although initial prevalence was significantly lower and fewer cases occurred during follow-up.

**What are the implications for public health practice?**

Proactive testing of LTCF residents and staff members might prevent large COVID-19 outbreaks in LTCFs through early identification and timely infection prevention and control response.

## Footnotes

* Turnaround time to receive test results from COVID-19 testing sites in Fulton County during the study period ranged from 3 to 10 days.

† One LTCF in the preventive group was tested by Fulton County before the addition of testing support from the National Guard.

§ In both the preventive and response groups, fewer than 1% of staff members and residents eligible for testing on the day of the testing event declined.

¶ Symptom-based screening included temperature checks and a survey of symptoms consistent with COVID-19; staff member screening occurred at the beginning of every shift, and resident screening varied by facility, occurring 1–12 times per day.

** All SARS-CoV-2 infections identified in staff members of LTCFs in the preventive group were identified through facility-wide testing and were not linked to outbreaks in other LTCFs.

†† Centers for Medicaid & Medicare Services requirements apply to nursing homes but not to assisted living facilities, which are not Medicaid/Medicare certified.

§§ A shelter-in-place order for the state of Georgia was instituted on April 2, 2020. https://gov.georgia.gov/executive-action/executive-orders/2020-executive-orders.

¶¶ https://www.cdc.gov/coronavirus/2019-ncov/hcp/clinical-guidance-management-patients.html.
